# Access to Virtual Mental Healthcare and Support for Refugee and Immigrant Groups: A Scoping Review

**DOI:** 10.1007/s10903-023-01521-1

**Published:** 2023-07-05

**Authors:** Michaela Hynie, Anna Oda, Michael Calaresu, Ben C. H. Kuo, Nicole Ives, Annie Jaimes, Nimo Bokore, Carolyn Beukeboom, Farah Ahmad, Neil Arya, Rachel Samuel, Safwath Farooqui, Jenna-Louise Palmer-Dyer, Kwame McKenzie

**Affiliations:** 1 Department of Psychology, York University, Toronto, Canada; 2 Centre for Refugee Studies, York University, 4700 Keele St., Toronto, ON M3J1P3 Canada; 3 Department of Psychology, University of Alberta, Edmonton, Canada; 4 Department of Psychology, University of Windsor, Windsor, Canada; 5 School of Social Work, McGill University, Montreal, Canada; 6 Department of Psychology, University of Quebec in Montreal, Montreal, Canada; 7 School of Social Work, Carleton University, Ottawa, Canada; 8 School of Nursing, Western University, London, Canada; 9 School of Health Policy and Management, York University, Toronto, Canada; 10 Department of Family Medicine, McMaster University, Hamilton, Canada; 11 Counseling Psychology, Yorkville University, Fredericton, Canada; 12 Wellesley Institute, Toronto, Canada; 13 Division of Health Equity, CAMH, Toronto, Canada; 14 Department of Psychiatry, University of Toronto, Toronto, Canada

**Keywords:** Telepsychiatry, Telemedicine, Mental Health, Refugees, Immigrants, Virtual Mental Health, Healthcare Accessibility

## Abstract

Immigrant and refugee populations face multiple barriers to accessing mental health services. This scoping review applies the (Levesque et al. in Int J Equity Health 12:18, 2013) Patient-Centred Access to Healthcare model in exploring the potential of increased access through virtual mental healthcare services VMHS for these populations by examining the affordability, availability/accommodation, and appropriateness and acceptability of virtual mental health interventions and assessments. A search in CINAHL, MEDLINE, PSYCINFO, EMBASE, SOCINDEX and SCOPUS following (Arksey and O’Malley in Int J Soc Res Methodol 8:19–32, 2005) guidelines found 44 papers and 41 unique interventions/assessment tools. Accessibility depended on individual (e.g., literacy), program (e.g., computer required) and contextual/social factors (e.g., housing characteristics, internet bandwidth). Participation often required financial and technical support, raising important questions about the generalizability and sustainability of VMHS’ accessibility for immigrant and refugee populations. Given limitations in current research (i.e., frequent exclusion of patients with severe mental health issues; limited examination of cultural dimensions; de facto exclusion of those without access to technology), further research appears warranted.

## Introduction

While access to mental health services was a well-documented challenge for refugee and immigrant populations before COVID-19 in countries around the world, the pandemic involved multiple changes, most notably the restriction of in-person services, that reconfigured obstacles and possibilities of care. Refugees and asylum seekers report higher rates of PTSD and common mental health disorders relative to the general population, and among those living with mental health problems mental health service use is lower in migrant than non-migrant poplations [1, 2]. Immigrants and refugees’ underutilization of mental health services has been attributed to the wide range of barriers they encounter when accessing mental health services. At the individual level, they can face communication difficulties, lack of trust, confidentiality concerns, feelings of shame, linguistic barriers and limits in mental health literacy and knowledge of accessible services. Provider-level barriers can include a lack of cultural competence and a lack of available providers. At the level of the intervention there can be challenges in the equity in the efficacy of interventions across groups. System-level barriers can include a lack of information about available and appropriate care, financial barriers, and lack of access to childcare and/or transportation. [2–7].

During the COVID-19 pandemic, public health recommendations for physical distancing led to many mental health services transitioning to virtual delivery. Evidence suggests that virtual care is effective for treating a range of mental health conditions and may increase accessibility for communities with limited access to appropriate mental health services, such as rural and newcomer populations [8, 9]; but while effectiveness of virtual mental health services have been explored, little is known about refugee and immigrant populations’ access to virtual care. This scoping review was motivated by a desire to better understand how this transition might improve or hinder access to mental health services for vulnerable migrant populations, and if the impact might be greater for particular sub-populations (e.g., immigrants, refugees and asylum seekers).

Virtual mental health care services (VMHS), also described as digital, remote or tele-health services, can include health services delivered by text, voice or video on a telephone or computer, an on-line application, or other technologically enhanced remote services [10]. We will refer to them collectively as VMHS. While offering promising avenues by increasing convenience and access to the number and range of providers, such modalities could also widen health inequalities, selectively improving services only for those who already have better access [10]. Indeed, many studies also suggest that accessing virtual care has actually presented additional challenges for individuals and communities who may lack technology devices or technical literacy, have unreliable and unaffordable internet connections, have concerns about privacy and confidentiality, or face communication barriers through video platforms [11–15]. These challenges suggest that a major limitation in the use of VMHS for newcomer communities may be linked to their accessibility.

The goal of this scoping review was to apply a multidimensional framework in identifying factors that affect the accessibility of virtual mental health services for immigrants, refugees and asylum seekers globally, across multiple countries and settings. Drawing from Levesque et al.’s Patient-Centred Access to Healthcare model [16] (see also Thiede et al. [17]), our framework included the dimensions of appropriateness/acceptability, affordability, and availability/accommodation. The Levesque et al. model defines healthcare access in terms of Approachability, Acceptability, Availablity and Accommodation, Affordability and Appropriateness, from the perspective of the service and the clients, respectively. This framework for evaluating access to healthcare was selected because of the emphasis on client-centred barriers and facilitators, which may better highlight the unique experiences of immigrants and refugees relative to other populations. Although the Levesque model does not explicitly refer to structural factors, a detailed analysis of how access is related to client needs and resources and the ways in which services are actually provided can uncover underlying structural factors that shape and limit mental health service accessibility.

Following the Levesque et al. model, we defined appropriateness as the fit to the clients’ needs, including language of service. Acceptability refers to the nature of service and how well it is perceived by the users, including cultural appropriateness. However, it should be noted that the issues of appropriateness and acceptability are clearly intertwined given the fact that newcomer communities often need services to be provided in a linguistic and culturally appropriate/safe manner for them to accept and access the service. Therefore, we consider these two dimensions together. Affordability refers to the fit between the cost of using services and a person's ability to pay, where the cost could be direct (e.g., provider fees) or indirect (e.g., internet fees). Availability/accommodation addresses physical accessibility of services, in this case, the extent to which virtual services are provided in a format that can actually be utilized by the intended user (e.g., literacy challenges).

## Methodology

We conducted a scoping review from November 2020 through October 2021, following adherence to the five general stage protocols recommended in the work of Arksey & O’Malley [18].

### Stage 1: Identifying the Research Question

The research question for the current scoping review was “What primary research exists that examines the accessibility (affordability, availability/accommodation, appropriateness and acceptability) of virtual mental health service delivery to specific populations or individuals who no longer reside in the country of their birth (i.e., immigrants, refugees and asylum seekers)?” The search utilized a range of terms for non-native born populations to capture articles published in different countries and with different migrant populations (see Appendix 1 for complete list). Efficacy was also addressed in this review because most published VMHS studies that focused on the assessment of service efficacy often included an assessment of accessibility.

### Stage 2: Identifying the Relevant Studies

Based on consultations with academic librarians, we began by constructing an initial Boolean search string. Boolean search is an algorithmic function applicable to many database search programs. It employs logical operators (e.g. AND, OR, NOT) and keyword terms to form logical search strings. When entered into a database, a single string allows for multiple permutations of keyword terms to be run simultaneously through system interfaces. The logical operators can either expand or limit those permutations, based on the nature of the desired query.

Following entry of the initial search string, we then ran multiple test searches, each time reviewing top results for additional search terms which could be incorporated into the existing string. Our final search string was an amalgam of three necessary focal areas of content. These included:

1. Themes related to psychiatric and/or psychological care or service.
2. Themes related to client populations who were either immigrants or refugees
3. Themes related to service delivery that included the specific use of technological mediums and/or devices (i.e., software and/or hardware)

We ran our finalized string through CINAHL, MEDLINE, PSYCINFO, EMBASE, SOCINDEX and SCOPUS, in December 2020 through October 2021. Each search was limited to title, abstract and keyword hits only, and only available, peer-reviewed, English language, full articles were assessed. Apart from limiting the search to English language text only, no further steps were taken that might intentionally limit the publication dates or geographic boundaries of the results. For our purposes, we hoped to capture a broad picture of various virtual mental health services as provided to immigrant, asylum seeker and/or refugee clients under a range of settings and conditions. A copy of the finalized keyword search string is presented in Appendix A.

### Stage 3: Selecting Studies

Combined results from the six main database searches captured 2526 abstract records for review. Over the course of the project, 35 additional abstract records were added, sourced from either Google Scholar or from references in relevant systematic reviews. The resulting 2561 abstract records were imported into Covidence, a free software platform designed for assisting in systematic review protocols [19].

Of the original 2561 abstracts, 1360 were eliminated as duplicates, leaving 1201 candidates for abstract screening. Six team members performed independent screenings of all remaining abstracts, wherein each document was reviewed by two or more separate researchers for inclusion. Inclusion criteria were:

A. A peer-reviewed product of a study reporting original research
B. Dealt specifically with either an immigrant or refugee population
C. Examined a psychiatric or psychological intervention (or assessment) where a substantial proportion of the intervention involved the use of technological devices and/or media
D. Provided either formal evaluations of intervention outcomes and/or reported on perspectives gathered from frontline service providers and/or their clients regarding the service.

Interrater reliability for abstract screening was 89.4%. In cases where initial reviewer assessments were in conflict, the original reviewers consulted with one another, offering rationales for their findings and subsequently deliberating the assessment. In cases where this process did not result in consensus, a third member of the review team was tasked with arbitration to resolve the conflict.

**Fig. 1:**
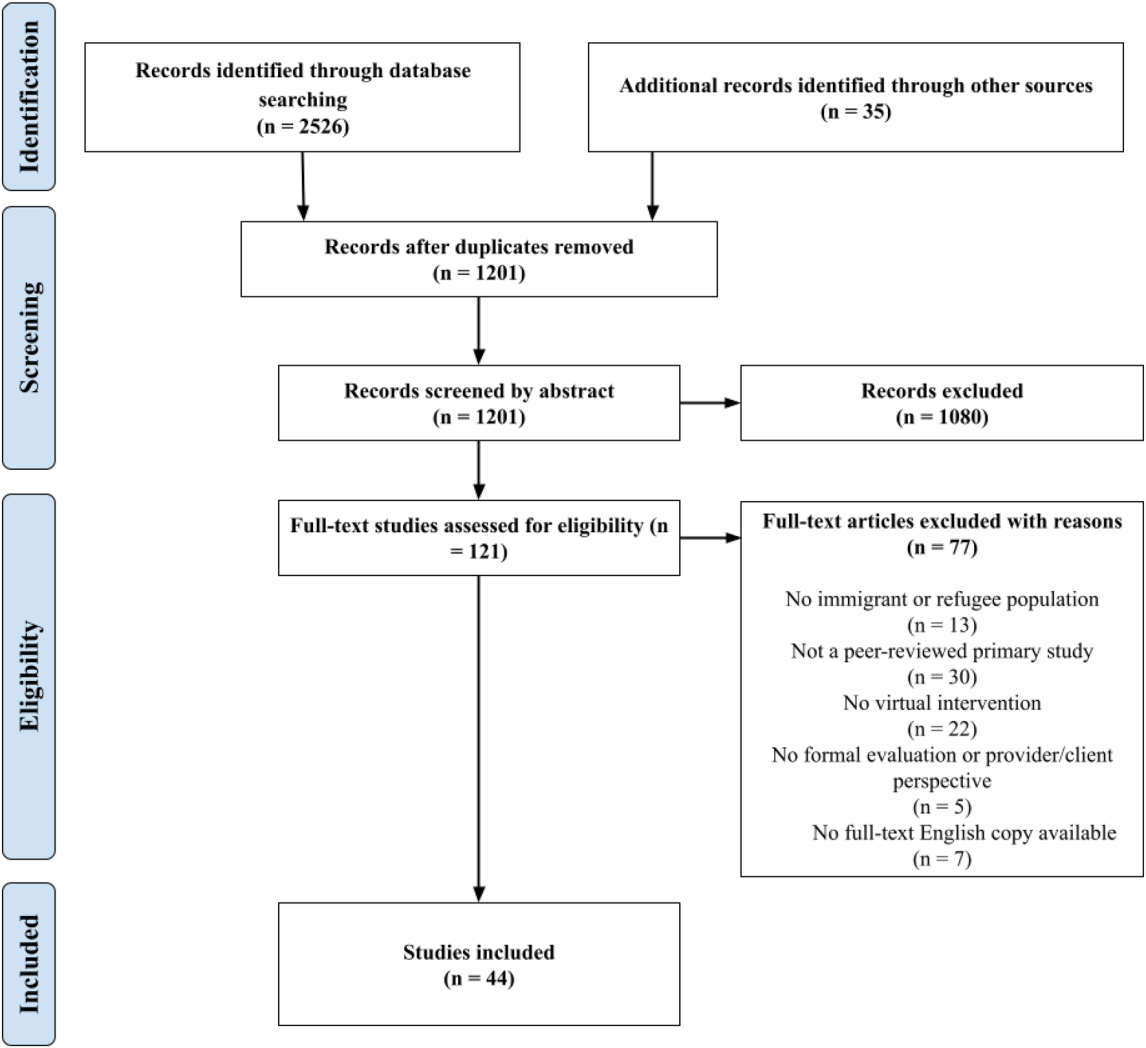
Study selection flow diagram

Abstract screening reviews for the 1201 candidate documents ultimately yielded 121 potential studies. The same six-member team then performed independent full-text reviews, where two-member consensus was required for a study to be graduated to the data extraction phase. At the end of this process, 44 relevant papers were promoted to the data extraction phase. However, these included papers reporting on the same interventions or even the same study. Two studies reported a different analysis on the same data from a text- and phone-based intervention, with one comparing intervention and control groups [20] and the other analyzing data only from the intervention group [21]. There were two follow-up studies on a tablet-based intervention [22]. The study published in 2017 was a qualitative interview study [23] with a subset of patient participants and the other, published in 2016, was a mixed methods study with perspectives of providers gathered via qualitative interviews, and analysis of quantitative data from the parent trial for the intervention arm for patients’ perspectives on the use of the tablet screening tool [24]. The was also a qualitative interview study [25] with participants who had completed an on-line iCBT intervention [26]. Thus, there were only 40 unique interventions/programs but all 44 papers are described here. A study selection flow diagram summarizing the process is presented in Fig. 1.

### Stage 4: Charting the Data

We created a tailored data entry form specific to our research question. The final draft of the form consisted of 39 separate fields covering several major areas of interest. These included: specific quantitative and qualitative aspects of the study design; major findings, outcomes, and descriptions of the study; particular elements noted concerning the technologically-mediated aspects of the interventions; and any barriers and/or facilitators mentioned with regard to the interventions. Data from each study was extracted separately by two independent reviewers, who later met to discuss and ratify entries for the finalized populated forms.

### Stage 5: Collating, Summarizing and Reporting the Result

Data from the finalized populated forms were then examined for recurring themes, identifying instances where there appeared to be conflicting results or outcomes, and reporting any obvious gaps in any of the chosen studies. In keeping with the Arksey and O’Malley [18] framework, a narrative account of the findings was created and is presented in terms of major themes below.

## Results

A brief narrative summary of the types of programs and users is provided in the sections below, followed by an analysis of the factors affecting accessibility. Furthermore, a summary of all the papers and the corresponding types of programs and users is presented in Table 1, *Study description, mental health condition(s) addressed and virtual modality of service delivery*.

**Table 1:** Study description, mental health condition(s) addressed, virtual modality of service delivery, and accessibility

References	Author	Study design	Sample population	Mental health condition(s) addressed	Virtual modality of service delivery	Any in-person elements?	Appropriate/acceptable	Affordable	Available/accommodating
Web-based self-paced									
[34]	Abi Ramia et al.	Qualitative study; Cultural adaptation	Syrian, Palestinian and Lebanese immigrants, refugees and residents in Lebanon	Depression	Self-paced web-based treatment with telephone support	No (the intervention is internet-based, with e-mail/messaging support but the participants were testing the cultural adaptation in focus groups or key informant interviews)	Cultural & linguistic adaptation Users valued anonymity Intervention rated as helpful and trusted programs	NA	Inclusion criteria—minimal literacy level
[39]	Burchert et al.	Qualitative study; Cultural adaptation	Syrian refugees in Germany, Sweden & Egypt	Depression	Self-paced web-based (phone or computer) CBT	Yes (the e-mental health intervention includes a guidance model which can be a human helper)	Cultural & linguistic adaptation—WHO e-mental health adaptation (Step by Step) in Arabic Intervention rated as helpful and trusted programs	NA	Technical literacy and problems with internet access were barriers
[40]	Davidson et al.	Single-arm design; Feasibility study	Immigrant male taxi drivers in Australia	General mental health	Self-paced web-based knowledge and self-help	No	No culture/linguistic modifications—only in English 2/3 of people endorsed recommending website to others	Inclusion criteria—owning devices—access to internet	NA
[44]	Eylem et al.	Randomised controlled trial; Feasibility study	Migrants of Turkish, Kurdish and Turkish Cypriot background in Netherlands and UK	Suicidal thoughts, depression, worrying, self-harm behavior and suicide attempt (SASH) and Acculturation	Self-paced web-based iCBT	No (mental health coaches provided on-line feedback to users and telephone interviews with subset of participants for usability feedback)	Cultural & linguistic adaptation-intervention provided in English, Dutch and Turkish Participants had contradictory opinions on acceptability of the intervention	NA	NA
[36]	Kanekar et al.	Randomised controlled trial; Pilot study	East Asian students in the USA	Well-being in general	Self-paced web-based course	No (but reminder emails were sent to users)	No culture/linguistic modifications—English only Large drop out rate	NA	
[38]	Kayrouz et al.	Single-group open trial; Feasibility study	Arab immigrants in Australia	Anxiety, depression, distress and disability	Self-paced web-based CBT	No (but additional online communication between therapist and user)	Linguistic adaptation—English only—some core words translated into Arabic Participants valued the anonymity Intervention rated as helpful and trusted program	NA	No need to travel
[63]	Kayrouz et al.	Single-group open trial; Pilot study	Arab immigrants in Australia	Anxiety and depression	Self-paced web-based knowledge and self-help	No (with automated emails to reinforce and summarize key skills)	Linguistic adaptation English only but key terms translated into Arabic Intervention rated as helpful and trusted program High dropout rate; low completion rates; low frequency of use	NA	Needed to have reliable internet access
[35]	Kayrouz et al.	Retrospective uncontrolled observational cohort study design	Immigrant adults in Australia	Anxiety and depression	Self-paced web-based CBT	No	No culture/linguistic modifications—English only	Needed to have reliable internet access	NA
[25]	Lindegaard, Kashoush, et al.	Qualitative study	Immigrants and refugees from Arabic-speaking countries in Sweden	Anxiety, depression, sleep problems, stress, worry and rumination, traumatic memories and emotion regulation difficulties	Self-paced web-based iCBT	No (the intervention on-line, initial clinical interview by phone, weekly feedback from therapists by on-line messages)	Cultural & linguistic adaptation-intervention provided in Arabic Lack of felt connection with the therapist due to the virtual nature of intervention	NA	Technical problems—difficulty logging into platform
[26]	Lindegaard, Seaton et al.	Randomised controlled trial; Pilot study	Refugees and immigrants from Arabic-speaking countries in Sweden	Depression, anxiety and stress	Self-paced web-based iCBT	No (facilitators also provided on-line feedback to users weekly)	Intervention offered in Arabic		
[41]	Nygren et al.	Randomised controlled trial	Kurdish immigrant men in Sweden	Depression, anxiety and insomnia	Self-paced web-based CBT	No (screening by phone or e-mail; all intervention on-line platform with videos and follow up messaging)	Tools translated into Kurdish	NA	Required a computer and internet connection
[33]	Ospina-Pinillos et al.	Qualitative study; Cultural adaptation	Spanish speaking Australians	General mental health	Web-based screening tool and personalized wellness program	No (development in person, user-testing virtual but observed in-person)	Co-design with community and health care providers to make culturally appropriate; use of Spanish diagnostic protocols Intervention rated as helpful and trusted program	Inclusion criteria—owning devices—access to internet	Technology platform—service should be accessible via smartphone (some students did not have desktop, laptop or tablets)
[37]	Ünlü Ince et al.	Randomised controlled trial; Summative evaluation	Turkish migrants in the Netherlands	Depression	Self-paced web-based problem-solving therapy	No (but users received feedback via weekly emails)	Cultural & linguistic adaptation—translated in Turkish Intervention rated as helpful and trusted program High dropout rate; low completion rates; low frequency of use	Inclusion criteria—owning devices—access to internet	NA
Computer video based									
[45]	Changrani et al.	Randomized controlled design. Feasibility study	Spanish speaking immigrant women in the USA	Depression, coping and quality of life	Computer on-line peer support	Yes (in-person technology training offered by bilingual facilitators)	Linguistic adaptation—Spanish	Provide dial up internet access—limited landline access; exceeded access plans	Provide assistance for people with technical difficulties Difficulty receiving support as could not be on phone and internet at same time
[46]	Jang et al.	Single group study; Feasibility study	Korean senior immigrants in USA	Depression	Computer video CBT	Yes (project manager brought and set up equipment, available for additional support)	Linguistic adaptation. Intervention rated as helpful and users trusted program	Not mentioned	Technical difficulties Hearing challenges
[50]	Mucic	Single group post-test only; Feasibility study	immigrants, refugee claimants and refugees in Denmark	Mental health in general	Computer video therapy	Yes (technical support on site of remote service)	Linguistic adaptation—therapy provided in clients’ first language Intervention rated as helpful and trusted program. Linguistic and cultural appropriateness compensated for technical challenges	In office program—all equipment set up at the site	Provide services in office where technical support available
[51]	Mucic	Single group post-test only; Feasibility study	immigrants, refugee claimants and refugees in Denmark	Psychiatric disorders	Computer video therapy	Yes (technical support on site of remote service)	Therapists from same culture and preferred language. Intervention rated as helpful and trusted program	In office program	Provided services in office where technical support available Reduced wait times and travel time
[49]	Pandya	Randomized controlled design	South Asian immigrant couples in the USA	Immigration stress and couple relationship	Computer video spiritual couples counseling	No	No culture/linguistic modifications	NA	Computer with internet connection or smartphone/tablet with wifi connection required
[47]	Ye et al.	Mixed methods design; Feasibility study	Korean immigrants in USA	Anxiety, depression and adjustment	Computer video therapy	Yes (technology set up and support on-site, follow up on clinical recommendations)	Cultural & linguistic adaptation—bilingual culturally competent therapists Therapeutic relationships difficult to establish Anonymity/privacy High level of acceptance	NA	Provided assistance for people with technical difficulties
[48]	Yeung et al.	Randomised controlled trial	Chinese immigrants in the USA	Major depression	Computer video therapy	Yes (technical support on site of remote service)	Cultural & linguistic adaptation through bicultural therapist	Cost effectiveness—availability of low-cost Webcams and free Web-based Skype software	Concern with security of platform (skype)
[62]	Zheng and Gray	Case series; Feasibility study	Chinese immigrants in the USA	PTSD and depression	Computer video therapy	Yes (technical support on site of remote service)	Therapists from same culture and preferred language Intervention rated as helpful and trusted program	NA	Video-conferencing based technology—allowed for rural participants
Telephone based programs									
[27]	Bayne et al.	Mixed-methods design	Latin American refugee claimants in the USA	Anxiety, depression and PTSD	Telephone assessment	Yes (interpreters in room with claimants)	No culture/linguistic modification Providers felt telephone was advantage	N/A	N/A
[31]	Jakobsen et al.	Diagnostic test validity	Asylum seekers mainly from Afghanistan & Somalia in Norway	Anxiety, depression and PTSD	Diagnostic psychiatric interview over the telephone	Yes (technological support and interpreters were present during self-administered screening tool)	Linguistic adaptation-questionnaires in participant languages (Dari, Somali, and Pashto)	Computer-based assessment is cost effective	NA
[44]	Mishori et al.	Qualitative study; Feasibility study	Asylums seekers in migrant encampments in Mexico (clinicians in the USA)	Psychological evaluations	Skype and WhatsApp evaluations	Yes (technical support always present)	Linguistic adaptation Therapeutic relationships difficult to establish	Program offered in office with available technology	Technical difficulties Provide services in office where technical support available
[54]	Serafini et al.	Observational study	Undocumented migrants from Hispanic origin in the United States	Not specified	Telepsychiatry over the telephone and WhatsApp	No (but many had transitioned from in-person to telephone services due to COVID-19 pandemic with same providers)	Linguistic adaptation	N/A	N/A
[55]	Walker et al.	Mixed methods design; Formative evaluation	Sudanese, Afghan, Burmese refugees in Australia	Well-being	Telephone peer support	Yes (in-person trainings were provided to the users)	Same culture (peer support), preferred language (translated materials). Satisfaction not reported	Free flat-rate phone plan provided for participants	Phone based-ease of use, familiarity of technology
[56]	Wollersheim et al.	Qualitative study; Formative evaluation	South Sudanese refugees in Australia	Trauma, settlement challenges	Telephone peer support	No	Linguistic adaptation—Support provided by members of same community. Readily accepted	Provide phone vouchers—amount given not always sufficient	Phone based-ease of use, familiarity of technology
[53]	Wetter et al.	Randomised controlled trial	Latin American immigrants in the USA	Smoking cessation	Telephone counseling	No	Cultural & linguistic adaptation—Spanish speaking service	Cost-effective. Covered costs	Phone widely accessible—85% Hispanic households have phones Addressed barriers to receiving treatment—transportation, time commitment, child care, and language
[57]	Zehetmair et al.	Qualitative study; Summative evaluation	Refugee claimants in Germany	PTSD, depression and distress	Self-paced phone-based audio guided imagery	Yes (included a face-to-face introductory session)	Linguistic adaptation—provided in preferred language Difficulty finding private locations in shelter to do audio relaxation Intervention acceptable but would prefer in-person	Inclusion criteria—owning devices—access to internet	Technical difficulties—with audio files
[52]	Zhu et al.	Randomised controlled trial	East Asian immigrant adults in USA	Smoking cessation	Telephone counseling	No	Cultural & linguistic adaptation—counselling protocol translated into Chinese, Korean, and Vietnamese	NA	NA
Text-based/e-mail									
[59]	Chiu et al.	Mixed methods design; Usability study	East Asian immigrant caregivers of Alzheimers patients in Canada	Caregiver burden, distress and depression	Web-based knowledge and e-mail support	No	Linguistic adaptation—caregiver information booklet and emails in language of choice (English or Chinese) High dropout rate; low completion rates; low frequency of use	NA	Difficulty remembering passwords and location of web address
[21]	García et al.	Quasi-experimental design	Immigrant women in Spain	Depression	Text messages-based CBT	Yes (face-to-face therapy and text messages)	Linguistic adaptation-offered in Spanish Intervention rated as helpful and trusted program	Problems with pre-paid phones when available credit was spent	Phone based—challenge for older women; unsure if this was due to technology or hearing difficulties
[20]	García et al.	Quasi-experimental design	Immigrant women in Spain	Depression	Personalized text messages with telephone therapy	Yes (face-to-face and remote therapy with text and telephone calls)	Linguistic adaptation Intervention rated as helpful and trusted program	Inclusion criteria—owning devices—access to internet	Phone based—challenge for older women; unsure if this was due to technology or hearing difficulties
[32]	Tomita et al.	Single group study; Feasibility study	Refugees mainly originating from Zimbabwe, Democratic Republic of Congo residing in South Africa	Depression	SMS (Short messaging system)-based assessment using mobile phones	No (but completed screening in clinic)	Linguistic adaptation—Intervention provided in English and Arabic Intervention rated as helpful and trusted program	NA	Phone based-ease of use, familiarity of technology
Touch screen/tablet based									
[22]	Ahmad et al.	Randomised controlled trial	Immigrants mainly originating from Latin America, South Asia and Africa or the Middle East in Canada	Major depression, generalized anxiety, PTSD and alcohol abuse	Touch-screen interactive computer-assisted client assessment survey (iCCAS)	No (self-administered screening tool but results used to inform subsequent in-person meeting with clinician)	Linguistic adaptation-intervention provided in English and Spanish Intervention rated as helpful and trusted program	NA	
[28]	Ahmad et al.	Randomised controlled trial	Chinese immigrants in Canada	Anxiety, depression, PTSD, and alcohol addiction	Tablet based assessment survey called iCCAS	No (but generates report to be reviewed in person with clinician at clinic)	Linguistic adaptation-translated into Mandarin and Cantonese Participants valued anonymity Intervention rated as helpful and trusted program	NA	Touch-screen survey offered in primary care clinic
[24]	Ferrari et al.	Follow-up mixed methods study of Ahmad et al. 2017 RCT: (i) provider qualitative interviews, (ii) 2ndry analysis of intervention arm (exit survey)	Almost all participants were immigrants in Canada	Major depression, generalized anxiety disorder, PTSD and harmful alcohol drinking	Tablet-based, touch-screen survey called iCCAS	No (self-administered screening tool but results used to inform subsequent in-person meeting with clinician)	Linguistic adaptation-intervention provided in English and Spanish	NA	Clients perceived as beneficial. Not sure about privacy and interaction barriers
[23]	Ferrari et al.	Follow-up study of Ahmad et al. 2017 RCT: Case study design with embedded units	Immigrants from Jamaica, Columbia, Iran, UK, Chile, Mexico in Canada	Major depression, generalized anxiety disorder, PTSD and harmful alcohol drinking	Touch-screen interactive computer-assisted client assessment survey called iCCAS	No (self-administered screening tool but results used to inform subsequent in-person meeting with clinician)	Linguistic adaptation-intervention provided in English and Spanish	NA	NA
[30]	Morina et al.	Randomized cross over design; Summative evaluation	Refugees and claimants in Switzerland	Psychiatric disorders	Tablet based assessment survey	No (but completed in clinic, and also completed paper-version of survey for comparison)	Available in multiple languages	NA	Inclusion criteria—digital literacy (able to use touch screen tablet) Literacy challenges overcome with audio presentation of items
[29]	Willey et al.	Qualitative study; Feasibility study	Afghan, Burmese, Indian, Vietnamese refugee and immigrant women in Australia	Perinatal depression	Tablet based assessment survey	Yes (interpreters available in clinic; midwives reviewed report with users)	Linguistic adaptation—interpretation provided where necessary; tool in English Answering questions in own language provided privacy and comfort	NA	Stand alone audio presentation and translation of materials into needed languages
Game-based interventions									
[60]	Holmes et al.	Single group study; Feasibility study	Refugees mainly originating from Syria and Iraq in Sweden	Psychological trauma, intrusive memories and concentration disruption	Computer gameplay using Tetris App	No (self-administered, but additional materials distributed in person by researchers)	Linguistic adaptation—intervention provided in English and Arabic Intervention rated as helpful and trusted program	NA	Smart phones always accessible
[61]	Quackenbush and Krasner	Case study	Libyan refugee (therapist in USA)	Anxiety and depression	Computer avatar text-based avatar therapy	No	No culture/linguistic modifications—English Virtual psychotherapy acceptable—as in-person therapy was not wanted, due to expense and social reasons		Concern with security of platform (skype)
[42]	Röhr et al.	Randomised controlled trial	Syria refugees in Germany	PTSD	Sanadak game available for Android/iOS App	No (intervention was a self-administered but screening and data collection (baseline, post-intervention, 4 week follow-up) were conducted in person)	Linguistic adaptation-intervention provided in Arabic App usability described as very good	Not cost effective—not effective for stand alone therapy	NA
[59]	Sirin et al.	Random controlled trial	Syrian refugee children in Turkey	Despair and cognitive functioning	Computer game	Yes (supervision and technical support in computer classroom)	Linguistic adaptation; Teachers had Turkish and Arabic materials Intervention rated as helpful and trusted program	Cost-efficient—limited resources required Specialized mental health provider not required	Use of a computer lab—program downloaded

Three studies were cultural adaptations of programs [31, 32, 37], several were described as feasibility or pilot studies [20, 22, 28, 36, 38, 42, 44–47, 50, 51, 60, 62], one was described as a usability study [59] and two appeared to be formative evaluations [55, 56]. Some of the feasibility/pilot studies were randomized controlled trials, but the majority of randomized controlled trials were concerned with effectiveness as well as acceptability [22, 25, 28, 30, 34, 35, 39, 41, 43, 48, 49, 52, 53, 60]. A small number of other designs were also represented (see Table 1).

### Nature of Intervention

The majority of the papers described a mental health intervention. However, ten papers described nine unique diagnostic assessment studies or screening tools [22, 23, 27–32] and two described the development, adaptation and/or user-testing of a screening tool and/or interventions [33, 34].

### Modality of Delivery

Modalities were classified by the *level* of in-person contact and *type* of technology involved. In-person contact varied across interventions, from fully virtual stand-alone programs that required no in-person contact to hybrid programs with in-person clinical follow up combined with remote therapy. Between these end points were a number of programs that offered virtual care with minimal in-person engagement for technical support, orientation to the intervention, booster sessions or check-ins at a later date.

The most frequent method of delivery across all studies was through stand-alone, self-paced web/mobile phone applications [34–45], the majority of which utilized self-help interventions based on iCBT, or problem-solving (see Table 1).

Video calls was the second most popular delivery modality. The majority of these interventions provided individual therapy or counseling using computer video connections, although one was used for psychological assessment [46] and one was a peer support program [47]. In some cases these services were provided in users’ homes [48–51], in other cases in designated offices for that purpose [46, 52, 53]. The majority of these interventions had in-person elements. For example, there were additional supports available with staff who provided an initial face to face session [50], provided in-person training on the technology [47, 49], and/or were available during the intervention to provide ongoing technical support to the client [46, 51, 53]. Some were a hybrid model where local staff would follow up with clients to support the clinical and treatment recommendations offered by the online therapist/counselor [49, 50, 52, 53]. The sole study of a stand-alone video-based program focused on a spiritual counseling program [51].

Phone interventions using mobile phone or landline technology and audio-only interactions tended to involve technology only as stand-alone services. Stand-alone programs included two phone-based addictions counseling programs for smoking [54, 55], one telepsychiatry program [56] and one telephone mental health assessment program [27]. Two initiatives by the same authors offered phone-based peer support [57, 58] where newcomer women were trained to provide support networks utilizing phones to stay in touch, and included in-person engagement in the training for the peers. One additional smart phone-based intervention was a self-paced, audio-led relaxation guide for managing distress that included an in-person, two-hour training and orientation session [59].

Tablet-based touch screen interventions included mental health assessment surveys that could be stand-alone [22, 28, 30], implemented in-person by a staff member through an interview [29] or in the presence of staff in a clinic, or self-administered, but intended to be a hybrid approach with a subsequent in-person follow-up assessment [22, 28]. The latter intervention, for example, generated reports for the clinician and client to be used for discussion with clinicians immediately after the survey/report.

Four studies used text-based messages either through phone/SMS [20, 32, 60] or e-mail [60]. Most combined other elements such as self-paced web-based information [60]. One that provided SMS text-based psychosocial therapy based on CBT was explicitly hybrid, with an in-person 30-min session at the beginning and then three brief phone check-ins through the course of the intervention [21]. The others appeared to be stand-alone psychosocial therapy [32] or psychosocial support [60].

An element of ‘gamification’ was present in several interventions. One employed a test of different computer games as educational/psychosocial interventions for refugee children in computer labs at their school [61], where staff were present in the room with the children to supervise and provide technical support. One was a stand-alone mobile phone-based game app for young refugees that used a Tetris-like game to help users control intrusive thoughts of trauma [62]. One case study described the use of avatars for stand-alone text-based psychotherapy via the internet [63].

### User Characteristics and Presenting Mental Health Concerns

While several interventions focused specifically on refugees or asylum seekers (See Table 1) [27, 31, 32, 39, 43, 56–59, 61–63], a larger proportion of the papers focused on initiatives involving immigrants [20, 22–24, 28, 36, 38, 40, 41, 47–51, 54, 55, 60, 64, 65]. One study looked at East Asian international students in the USA [36]. The remaining initiatives focused on migrants in general, often from specific ethnocultural or linguistic groups, with mixed migration pathways: Spanish-speaking Australians, [33]; immigrants, refugees and refugee claimants in Denmark [47]; Afghan, Burmese, Indian and Vietnamese refugees and immigrants in Australia [29]; refugees and refugee claimants in Switzerland [30]; Syrian, Palestinian and also Lebanese origin in Lebanon [34]; immigrants and refugees from Arabic-speaking countries in Sweden [42]; migrants of Turkish, Kurdish and Turkish Cypriot background in the Netherlands and UK [44]; refugees and immigrants from Arabic-speaking countries in Sweden [45]).

Exclusion criteria in several studies eliminated participants who had serious mental health conditions including psychosis, very severe depression, anorexia, bipolar disorder, or a risk of suicide [20, 37, 41–43, 45, 48–50, 52, 59, 65] and in one case those who were disoriented or expressed aggressive behaviour [53]. The remaining programs had no exclusion criteria related to mental health or illness.

In terms of the clinical characteristics of users, the majority of VMHS focused on clients’ symptoms of depression [20, 29, 32, 34, 37, 39, 46, 48, 58] or depression in combination with anxiety [26, 35, 38, 41, 61, 63] or with PTSD/trauma [57, 62]; anxiety and PTSD [25, 27, 31]; anxiety, PTSD and substance use (primarily alcohol use [22–24, 28]; or caregiver burden [58]. Two interventions addressed only PTSD/trauma [42, 60], and two addressed substance use (smoking cessation [50, 53]. Some focused on adjustment or acculturation in combination with couple relationships [49], PTSD/trauma [62] or depression and anxiety [42, 45, 54]; others on general clinical disorders [30, 49]; and five focused on general mental health or well-being [33, 36, 40, 50, 55]. Self-paced, web-based interventions tended to focus primarily on depression or depression and anxiety, but with one exception [42]; these services were typically not intended to address more severe PTSD/trauma issues, and often involved elements of CBT. Touch screen/tablets were used primarily for screening tools to support in-person clinical visits and discussions with providers. Other modalities were used for a wide range of mental health and wellness concerns.

### Factors Affecting Access to Virtual Services for Immigrant and Refugee Populations

Table 1 summarizes data related to service appropriateness/acceptability, affordability, and availability/accommodation. These are described in more detail below.

#### Appropriateness/Acceptability

Interventions were generally rated as helpful and users mostly reported trusting the programs when client assessments were collected [20, 22, 28, 32–34, 37–39, 46, 50, 51, 54, 60, 62, 63]. However, some factors affecting accessibility emerged from the studies. One of the most promising aspects of virtual care for migrant populations is the ability to bridge the key cultural and language barriers by allowing migrant users to reach services and providers with language skills and cultural knowledge who may not be available in their own geographic locations [14]. Highlighting the critical importance of language, an SMS-based program to assess depression symptoms among refugees in South Africa showed that a major reason for the low utilization of the service was that the program was only offered in English [32]. We therefore looked specifically at what kinds of language and cultural adaptations were described in these programs. These were categorized as linguistic modifications [20, 22–24, 28, 31, 32, 42, 44–46, 50, 54, 56–60, 63], cultural modifications [33, 38, 62], combined cultural and linguistic modifications [26, 34, 36, 39, 41, 43, 47, 48, 52, 53], utilization of cultural- and ethnic-matching service providers with the clients’ preferred language (with no explicit mention of adaptation) [51, 55], or either no modifications or modifications not clearly explained or specified [25, 35, 36, 40, 49, 61]. In some cases, those reporting only translation may have also included cultural adaptation as part of the translation process, but this was not clearly stated. In video-calls, in particular, the emphasis was typically on care being provided by someone bilingual (typically from the same community) without detailing modifications. Articles involving phone and self-paced web interventions were even less likely to report any possible adaptations. Interestingly, cultural adaptations of a self-paced web CBT program for Turkish migrants in the Netherlands and the UK found that although the content of the program was culturally appropriate, the concept of self-help was not, suggesting that minor cultural adaptations may not be sufficient [43].

Appropriateness was also an issue in terms of severity of mental health issue. For both the users and those designing VMHS, virtual delivery has been found to be problematic for those with serious mental illness [50, 51]. Or, from a different angle, some users opted for virtual interventions because they felt that their mental health symptoms were not sufficiently severe to warrant visiting a therapist face to face [38].

Issues pertaining to privacy and anonymity were both enhanced and exacerbated in offering virtual services. Users of different self-paced stand-alone web-based programs reported preferring on-line services because they could be accessed anonymously, thereby avoiding the stigma associated with using mental health services [28, 34, 38]. But users of a self-paced audio-based relaxation intervention that was offered to refugees in Germany reported that they could not find quiet locations where they could use the intervention in the reception centres in which refugees and asylum seekers live [57]. In at least one study, some users of a tablet-based screening tool reported that they were unsure about how private their information was through VMHS [24].

Among those programs offering video call-based therapy, authors noted that users reported high rates of satisfaction. Some reported that therapeutic relationships were more difficult to establish [44, 47] but users reported that the benefits of having linguistically and culturally appropriate care compensated for challenges or dissatisfaction with the technology [50]. Video-based programs resulted in high completion rates though technical issues such as hearing impairments were reported as interfering with therapeutic interactions. However, some programs, particularly the self-paced ones, reported a wide range of dropout rates and completion rates, anywhere from 9% (e.g. [40]) to around 40% [26, 36, 37], with smaller samples typically having higher completion rates, as is typical for iCBT programs with other populations [63]. However, the reasons for withdrawing were typically not known since participants were not followed up if they withdrew. At the same time, self-paced programs had moderate to high satisfaction among those who participated, and users agreed that they reduced costs and stigma and that the programs taught them new coping techniques. In the self-paced audio relaxation program, users reported that the intervention was acceptable but they would have preferred in-person treatment [57], which is consistent with other research on VMHS [14].

Other appropriateness and accessibility issues that emerged included factors associated with clients’ individual characteristics or personal experiences. Clinicians reported that video assessment could be more difficult with clients who were avoidant or reserved because they shared less information and that facial expressions and other nonverbal cues were limited in this modality [44]. Moreover, a tablet-based assessment tool was found to have lower detection of mental health symptoms among those women who had lower levels of education. The authors suggested that health literacy, which often correlates with education levels, also plays a role in the effectiveness of these virtual tools; technological support alone may not make the tool useful if users cannot recognize and communicate symptoms [22]. Likewise, those with lower education levels reported less satisfaction with a video-therapy program [51]. In this same study, which provided the remote services in clinical offices, small confined spaces created discomfort and were thus inappropriate for those clients with a history of detention, such as those who experienced forced migration [51].

#### Affordability

Affordability of services was affected by the costs of technological devices (mobile phones, tablets, computers, and/or cameras), the cost of internet services in the home, or the cost of data plans for mobile phones. Some programs offered their services in offices, circumventing the issue of cost of equipment for clients or having sufficient bandwidth, while still providing access to remote providers [44, 50, 51, 62]. Other interventions addressed costs of virtual access by providing the users with the needed devices or providing funding for the services users required to participate [32, 45, 56]. For example, a phone-based peer support program for refugee women in Australia provided the women with phone vouchers to cover the cost of phone calls, but also noted that the amount provided was not always sufficient [56]. Similarly, a video-based on-line peer support program for Spanish-speaking immigrant women in the USA found that even though they provided participants with dial-up access for internet services, many had limited land-line access and participation exceeded their access plans [45]. The potential barrier of costs associated with VMHS was often not apparent in the studies because these studies specified having the necessary equipment or access to the internet as an inclusion criterion for participating in the studies [20, 33, 37, 40, 55].

#### Availability/Accommodation

Technical issues emerged as a barrier to availability, both in terms of the quality of the technology itself, and the challenges users faced with operating the technology, such as dropped calls, poor-quality video or corrupted audio files, or just unspecified “technical difficulties” reported by users [44, 46, 62]. In one study the authors discussed having concerns about the security of the platform they were using and whether the security was adequate for the delivery of mental health services [48].

Other relevant newcomer client factors, such as basic literacy or digital literacy, were often implied but not explicitly addressed in the studies because participants were selected on the basis of whether they were able to use the technology (e.g., able to use a touch-screen tablet, [30]) or had a minimal level of basic literacy [34]. In some projects without these exclusions, digital literacy emerged as an important barrier. A study of a self-paced e-mental health app for refugees reported that both access to technology and digital literacy were common challenges [32]. A Canadian study with immigrants who were caregivers reported that some participants struggled with remembering passwords for their log-ins to receive the email-based support, or with being able to locate the address of the web-based knowledge intervention [58]. A self-paced web-based CBT program for immigrants in Sweden reported that some struggled to log in to the platform [25].

Several projects included individuals who were available to provide assistance to participants if they had technical difficulties [22–24, 28, 45–47], or provided the services in community or clinical offices where there was technological support [44, 50, 51, 62]. This suggests that providing this support could be challenging if the service is intended to be delivered remotely, creating challenges for initiatives that are fully virtual; in one project, users accessed the internet using dial-up services and could not be on-line and receiving technological support by phone at the same time [45]. Collectively, one could interpret that these studies suggest that some in-person support may be necessary for addressing technological issues.

Benefits of phone-based interventions included the widespread use of home and mobile phones, ease of use, and users’ familiarity with phone communication [32, 55, 56]. Nonetheless, two papers describing a phone-based peer support program reported that older women in their sample were less likely to participate, although the authors did not indicate if this was due to discomfort with technology [20, 21]. Hearing difficulties emerged as a barrier to participation for older participants in another study [46]. Recognizing that there may be relationships between, for example, age and hearing suggests the need to consider intersectional aspects of accessibility beyond language and culture.

Offering mental health services in multiple modalities helped address some barriers to access, especially those related to challenges with literacy. Some tablet-based screening tools had options for audio presentation of the questions (e.g., [29, 30], circumventing the need to read the questions. The provision of interpretation for tablet-based screening tools offered in a limited number of languages raised issues of privacy that could make users of programs uncomfortable, further reinforcing the value of stand-alone audio presentation and translation of materials into needed languages [29, 51].

## Discussion

While comparing the accessibility of programs across so many different modalities and populations can be challenging, this current scoping review on access to virtual mental health care for immigrant and refugee populations reveals some important common facilitators and barriers. Many are consistent with those reported in general populations (e.g., [64]) but some are unique or more common in this specific group, as described below. Barriers to access that emerged in this review include individual level determinants of digital access, the nature of the program or intervention, and structural barriers associated with the larger social context in which the services are delivered.

### Intersectionality and Individual Level Barriers

In terms of individual level factors, these facilitators and barriers were not distributed equally across populations. Differences were seen in digital literacy, in the ability to afford and access technology, and in literacy more generally. However, constraining social and demographic conditions are likely to co-occur and to be more common in some migration pathways [66–68]. For example, older migrants face unique challenges that can include less digital literacy, lower fluency in the local languages, and some impairments in their visual and auditory abilities [68, 69]. Refugees and asylum seekers are also more likely than voluntary migrants to have elevated rates of serious mental health concerns, especially if they are still residing in situations of asylum [70, 71]. This may be a concern given that many studies did not include participants with serious mental health conditions and the appropriateness of virtual modalities in these situations has been questioned [50, 51]. Depending on their country of origin or asylum, they may have less technology access and lower levels of digital literacy [72]. These individuals are also more likely to have constraints on their financial means and physical space than other migrants, especially when migration policies restrict their access to employment and housing [73].

Migration experiences also shape preferences and comfort with VHMS. Asylum seekers and refugees are more likely than other migrants to have been imprisonned and/or tortured. Participants with these experiences were reported as being more concerned with issues of confidentiality when using interpreters and as presenting lower levels of trust in virtual settings [14, 74, 75]. Authors noted that these experiences were also associated with these participants’ discomfort or distress when receiving virtual services in small, confined office spaces [14, 74, 75]. These studies also highlight the importance of literacy and education more generally. The intersectional nature of these access barriers is important to consider, as those who may face the most significant barriers in accessing mental health services in general may also be the ones facing the greatest barriers in accessing services offered in virtual modalities.

### Strategies Facilitating Access to Virtual Services

Several issues emerged in these studies that raise questions about the generalizability of the findings and the sustainability of programs that required additional supports to be accessible. Many programs offered facilitators who would support clients’ use of the technology (e.g., [62]) and in some cases also to support clients with follow up on therapeutic interventions or intervene in case of emergencies (e.g., [56]). Most of these interventions also included in-person sessions to support the virtual interventions, including in-person introductions to the program and training in using the technology (e.g., [45, 57]). Many also had booster sessions part way through the program, suggesting that hybrid approaches may be the most accessible form of VMHS and may even be necessary for programs to be fully functional and accessible. Studies with inclusion criteria that excluded participants with limited literacy, language skills or serious mental health issues make it difficult to assess appropriateness and acceptability with the population as a whole. While patient safety may have made it necessary to exclude those who may be most at risk or may not be able to consent, this makes it difficult to assess the mental health conditions for which virtual programs would be appropriate. Those that did include people experiencing more serious mental illnesses did suggest that virtual care may not be appropriate in these cases [76, 77]. These hidden challenges may partially explain the “research to practice” gap that has been observed in the implementation of VMHS [78–80], and highlights that a one-size-fits-all approach to VMHS is unlikely to work in practice for various newcomer individuals and communities. Rather, VMHS needs to be tailored to target populations, local circumstances and available providers.

### Inclusion and Exclusion Criteria

Evaluating accessibility was also hindered by some missing information in the studies reviewed. Many studies did not explore or explain drop-out rates or the uptake of the intervention among possible participants, making it difficult to evaluate who found the interventions inaccessible or unacceptable [79]. Many studies also did not provide information about whether they used systematic cultural adaptation, allowing shared language and culture to stand in as cultural adaptation or actually using a range of cultural adaptation approaches (cf. [81]). Furthermore, this scoping review included studies on VMHS addressing well-being and mental health issues, from psychosocial well-being to PTSD, and some studies were unclear regarding how they defined well-being or mental health, making comparisons across studies challenging. Finally, the choice of specific technology for the intervention was often driven by pragmatic assumptions about the widespread use of a technological device (e.g., phone), without clinically based justifications or rationales. It was typically unclear if the chosen modality was the most appropriate intervention tool or why it was employed in relation to the clients’ mental health conditions and needs.

### Therapeutic Benefits and Challenges

While efficiency of virtual care was not the focus of this scoping review, many articles raised relevant points on therapeutic benefits and challenges that are worth mentioning. Hassan and Sharif [82] concluded from their systematic review of 14 randomized controlled studies of telepsychiatry interventions for refugees that virtual psychotherapeutic treatments are just as effective as traditional, in-person treatment modalities (cf. [2]). Recent studies conducted during COVID-19 have also noted positive attitudes toward and favorable uptake of VMHS by refugee clients and refugee-serving providers and stakeholders [11, 12, 14]. Effectiveness of the intervention was not a focus of the present scoping review, but could be considered relevant to the issue of appropriateness. Consistent with Hassan and Sharif’s general findings, several studies reported improvements in mental health conditions across studies with varying designs and quality (anxiety: [35, 41]; GAD-7: [37, 62]; depressive symptoms: [20, 21, 38, 41, 46, 48, 63]; depression [62]; PTSD: [59]; decreased general stress: [49, 57]; decreased acculturative or immigration stress: [55]; post-traumatic growth: [45]; smoking abstinence: [52]; a range of adjustment or well-being measures [40, 47, 56]; and social support related outcomes [55, 56]).

Despite reporting positive outcomes, some studies reviewed noted challenges with building a therapeutic alliance in virtual modalities [83–85]. Service providers that have not developed the skills needed to build trust and offer culturally appropriate care with immigrant and refugee populations [86–89] may find these challenges to be exacerbated in virtual settings [90]. While many users reported satisfaction with the services they received and found them effective, some noted challenges in reading body language, disruptions due to technological issues and user preferences for in-person care. These observations suggest that, going forward, virtual modalities would benefit from deliberate reflection and modification of therapeutic techniques to enhance therapeutic alliance [14, 91].

### Limitations and Future Research

The scoping review has some limitations that need to be considered. It did not assess the quality of the studies undertaken, which were represented by a range of methodologies, including RCTs, case studies, and studies focused on the development and usability testing of interventions. This review was also intended to be broad in scope and thus included studies using different virtual modalities, for various type of users (both general population and clinical populations), addressing a variety of mental health issues, with different immigrant groups residing in different countries, and surveying a wide spectrum of ways in which VMHS are being used for intervention and diagnosis. As a next step, a systematic review of VMHS focusing on specific modalities, mental health issues, populations and settings would be valuable in extending the current literature and our understanding about this important subject. These differences may inadvertently mask important cultural variations in the understanding of mental health and illness between and among newcomer groups as well as cross-cultural differences in the perceived appropriateness and acceptability of the different virtual modalities for different kinds of mental health and psychological conditions. The small number of studies on the gamification of mental health services also warrant added attention as a newer approach to services for some populations.

Lastly, a major issue and challenge for research on access to VMHS as a whole is that users who experience the most profound obstacles in accessing those services are unlikely to be included in such studies. Futher research exploring these issues is thus needed to assess accessibility in a more nuanced way, integrating clinical, cultural, and structural dimensions.

## Conclusion

The COVID-19 pandemic made VMHS a necessity, but in so doing opened up new opportunities for increased access to mental health care for various populations, including immigrants and refugees, and it seems likely that virtual approaches will continue to be promoted [14]. This scoping review suggests that the potential of virtual mental healthcare to reach underserved populations may not be achieved because of insufficient consideration of barriers for those already facing the greatest challenges in accessing care (e.g., those with limited language fluency, digital literacy or access to devices). This includes neglecting whether the additional supports required to make VMHS accessible (e.g., providing devices and financial support for phone or internet services) will be available in programs once they are beyond the testing and research phase, highlighting the importance of more implementation research. A number of common challenges in VMHS accessibility were identified across this diverse range of interventions and populations; however, this scoping review also identified unique barriers determined by systemic, contextual, clinical and personal characteristics for immigrant and refugee populations. Such obstacles warrant further attention. We propose that working with the intended user population on the planning and delivery of virtual mental health services will help increase accessibility for these populations, both now and in the future.

## Appendix

### Themes Related to Psychiatric and/or Psychological Care or Service

addiction OR anxiety OR bipolar OR counseling OR counselling OR depression OR “depressive disorder*” OR “dissociative disorder*” OR “mental disorder*” OR “mental health” OR “mood disorder*” OR “obsessive–compulsive disorder*” OR OCD OR “panic disorder*” OR “personality disorder*” OR postpartum OR “posttraumatic stress disorder*” OR “post-traumatic stress disorder*” OR “psychiatric care” OR “psychiatric therap*” OR “psychiatric treatment*” OR psychoeducation OR “psychological care” OR ‘psychological distress” OR “psychological therap*” OR “psychological trauma*” OR “psychological treatment*” OR “psychosocial support*” OR psychotherap* OR PTSD OR schizo* OR “stress disorder*” OR “substance abuse” OR “substance dependenc*” OR “substance withdrawal”

**AND**

### Themes Related to Client Populations Who Were Either Immigrants or Refugees

alien OR aliens OR “asylum seeker*” OR “displaced person*” OR DP OR escapee* OR exile* OR foreign-born OR foreigner* OR fugitive* OR immigrant* OR migrant* OR newcomer* OR non-native* OR refugee*

**AND**

### Themes Related to Service Delivery that Included the Specific Use of Technological Mediums and/or Devices (i.e. Software and/or Hardware)

app OR app-based OR digital OR “digital health” OR “digital psychiatry” OR “emental health” OR “e-mental health” OR “mobile-based” OR online OR phone-based OR “remote consultation*” OR teleconferenc* OR telehealth OR telemedicine OR telemental OR telephon* OR telepsychiatry OR telepsychology OR videoconferenc* OR virtual OR web-based

## References

[1] Satinsky E, Fuhr DC, Woodward A, Sondorp E, Roberts B (2019). Mental health care utilisation and access among refugees and asylum seekers in Europe: a systematic review. Health Policy.

[2] McKenzie K, Agic B, Tuck A, Antwi M (2016). The case for diversity: building the case to improve mental health services for immigrant, refugee, ethno-cultural and racialized populations.

[3] Byrow Y, Pajak R, Specker P, Nickerson A (2020). Perceptions of mental health and perceived barriers to mental health help-seeking amongst refugees: a systematic review. Clin Psychol Rev.

[4] Khanlou N, Haque N, Sheehan S, Jones G (2014). “It is an issue of not knowing where to go”: service providers’ perspectives on challenges in accessing social support and services by immigrant mothers of children with disabilities. J Immigr Minor Health.

[5] van der Boor CF, White R (2019). Barriers to accessing and negotiating mental health services in asylum seeking and refugee populations: the application of the candidacy framework. J Immigr Minor Health.

[6] Wohler Y, Dantas JA (2016). Barriers accessing mental health services among culturally and linguistically diverse (CALD) immigrant women in Australia: policy implications. J Immigr Minor Health.

[7] McKenzie K, Hansson E, Tuck A, Lurie S (2010). Improving mental health services for immigrant, refugee, ethno-cultural and racialized groups: issues and options for service improvement. Canadian Issues.

[8] Mental Health Commission of Canada. Advancing the mental health strategy for Canada [Internet]. Ottawa, ON; 2016. Available from: https://www.mentalhealthcommission.ca/wp-content/uploads/drupal/2016-08/advancing_the_mental_health_strategy_for_canada_a_framework_for_action.pdf

[9] Titov N, Hadjistavropoulos HD, Nielssen O, Mohr DC, Andersson G, Dear BF (2019). From research to practice: ten lessons in delivering digital mental health services. J Clin Med.

[10] Latulippe K, Hamel C, Giroux D (2017). Social health inequalities and eHealth: a literature review with qualitative synthesis of theoretical and empirical studies. J Med Internet Res.

[11] Benjamen J, Girard V, Jamani S, Magwood O, Holland T, Sharfuddin N (2021). Access to refugee and migrant mental health care services during the first six months of the COVID-19 pandemic: a Canadian refugee clinician survey. Int J Environ Res Public Health.

[12] Disney L, Mowbray O, Evans D (2021). Telemental health use and refugee mental health providers following COVID-19 pandemic. Clin Soc Work J.

[13] Gajarawala SN, Pelkowski JN (2021). Telehealth benefits and barriers. J Nurse Pract.

[14] Hynie M, Jaimes A, Oda A, Rivest-Beauregard M, Perez Gonzalez L, Ives N (2022). Assessing virtual mental health access for refugees during the COVID-19 pandemic using the levesque client-centered framework: what have we learned and how will we plan for the future?. Int J Environ Res Public Health.

[15] Jacobson C, DeYoung, J, Ibarra O. Barriers to virtual care access impacting already underserved communities. [Internet]. United States of Care.; 2021. Available from: https://unitedstatesofcare.org/barriers-to-virtual-care-access-impacting-already-underserved-communities/

[16] Levesque JF, Harris MF, Russell G (2013). Patient-centred access to health care: conceptualising access at the interface of health systems and populations. Int J Equity Health.

[17] Thiede M, Akweongo P, McIntyre D (2007). Exploring the dimensions of access.

[18] Arksey H, O’Malley L (2005). Scoping studies: towards a methodological framework. Int J Soc Res Methodol.

[19] Veritas Health Innovation. Covidence systematic review software [Internet]. Melbourne; Available from: www.covidence.org

[20] García Y, Ferrás C, Ginzo MJ (2020). Effectiveness of a psychosocial therapy with SMS in immigrant women with different degrees of depression. Soc Sci Basel.

[21] García Y, Ferrás C, Rocha Á, Aguilera A (2019). Exploratory study of psychosocial therapies with text messages to mobile phones in groups of vulnerable immigrant women. J Med Syst.

[22] Ahmad F, Ginsburg L, Dinca-Panaitescu S, Lou W, Shakya Y, Ledwos C (2017). Preconsult interactive computer-assisted client assessment survey for common mental disorders in a community health centre: a randomized controlled trial. CMAJ Open.

[23] Ferrari M, Shakya Y, Ledwos C, McKenzie K, Ahmad F (2017). Patients’ mental health journeys: a qualitative case study with interactive computer-assisted client assessment survey (iCASS). J Immigr Minor Health.

[24] Ferrari M, Ahmad F, Shakya Y, Ledwos C, McKenzie K (2016). Computer-assisted client assessment survey for mental health: patient and health provider perspectives. BMC Health Serv Res.

[25] Lindegaard T, Kashoush F, Holm S, Halaj A, Berg M, Andersson G (2021). Experiences of internet-based cognitive behavioural therapy for depression and anxiety among Arabic-speaking individuals in Sweden: a qualitative study. BMC Psychiatry.

[26] Lindegaard T, Seaton F, Halaj A, Berg M, Kashoush F, Barchini R (2021). Internet-based cognitive behavioural therapy for depression and anxiety among Arabic-speaking individuals in Sweden: a pilot randomized controlled trial. Cogn Behav Ther.

[27] Bayne M, Sokoloff L, Rinehart R, Epie A, Hirt L, Katz C (2019). Assessing the efficacy and experience of in-person versus telephonic psychiatric evaluations for asylum seekers in the U.S.. Psychiatry Res.

[28] Ahmad F, Wang J, Wong B, Fung WLA (2022). Interactive mental health assessments for Chinese Canadians: a pilot randomized controlled trial in nurse practitioner-led primary care clinic. Asia-Pac Psychiatry.

[29] Willey SM, Blackmore RP, Gibson-Helm ME, Ali R, Boyd LM, McBride J (2020). “If you don’t ask … you don’t tell”: refugee women’s perspectives on perinatal mental health screening. Women Birth J Aust Coll Midwives.

[30] Morina N, Ewers SM, Passardi S, Schnyder U, Knaevelsrud C, Müller J (2017). Mental health assessments in refugees and asylum seekers: evaluation of a tablet-assisted screening software. Confl Health.

[31] Jakobsen M, Meyer DeMott MA, Heir T (2017). Validity of screening for psychiatric disorders in unaccompanied minor asylum seekers: Use of computer-based assessment. Transcult Psychiatry.

[32] Tomita A, Kandolo KM, Susser E, Burns JK (2016). Use of short messaging services to assess depressive symptoms among refugees in South Africa: implications for social services providing mental health care in resource-poor settings. J Telemed Telecare.

[33] Ospina-Pinillos L, Davenport T, Mendoza AD, Navarro-Mancilla A, Scott EM, Hickie IB (2019). Using participatory design methodologies to co-design and culturally adapt the Spanish version of the mental health eclinic: qualitative study. J Med Internet Res.

[34] Abi Ramia J, Harper Shehadeh M, Kheir W, Zoghbi E, Watts S, Heim E (2018). Community cognitive interviewing to inform local adaptations of an e-mental health intervention in Lebanon. Glob Ment Health.

[35] Kayrouz R, Karin E, Staples LG, Nielssen O, Dear BF, Titov N (2020). A comparison of the characteristics and treatment outcomes of migrant and Australian-born users of a national digital mental health service. BMC Psychiatry.

[36] Kanekar A, Sharma M, Atri A (2010). Enhancing social support, hardiness, and acculturation to improve mental health among Asian Indian international students. Int Q Community Health Educ.

[37] Ünlü Ince B, Cuijpers P, van Hof E, van Ballegooijen W, Christensen H, Riper H (2013). Internet-based, culturally sensitive, problem-solving therapy for Turkish migrants with depression: Randomized controlled trial. J Med Internet Res.

[38] Kayrouz R, Dear BF, Johnston L, Gandy M, Fogliati VJ, Sheehan J (2015). A feasibility open trial of guided Internet-delivered cognitive behavioural therapy for anxiety and depression amongst Arab Australians. Internet Interv Appl Inf Technol Ment Behav Health.

[39] Burchert S, Alkneme MS, Bird M, Carswell K, Cuijpers P, Hansen P (2019). User-centered app adaptation of a low-intensity e-mental health intervention for Syrian refugees. Front Psychiatry.

[40] Davidson S, Fletcher S, Wadley G, Reavley N, Gunn J, Wade D (2020). A mobile phone app to improve the mental health of taxi drivers: single-arm feasibility trial. JMIR MHealth UHealth.

[41] Nygren T, Brohede D, Koshnaw K, Osman SS, Johansson R, Andersson G (2019). Internet-based treatment of depressive symptoms in a Kurdish population: a randomized controlled trial. J Clin Psychol.

[42] Röhr S, Jung FU, Pabst A, Grochtdreis T, Dams J, Nagl M (2021). A self-help app for Syrian refugees with posttraumatic stress (Sanadak): randomized controlled trial. JMIR MHealth UHealth.

[43] Eylem O, van Straten A, de Wit L, Rathod S, Bhui K, Kerkhof AJFM (2021). Reducing suicidal ideation among Turkish migrants in the Netherlands and in the UK: the feasibility of a randomised controlled trial of a guided online intervention. Pilot Feasib Stud.

[44] Mishori R, Hampton K, Habbach H, Raker E, Niyogi A, Murphey D (2021). “Better than having no evaluation done”: a pilot project to conduct remote asylum evaluations for clients in a migrant encampment in Mexico. BMC Health Serv Res.

[45] Changrani J, Lieberman M, Golant M, Rios P, Damman J, Gany F (2008). Online cancer support groups: experiences with underserved immigrant Latinas. Prim Psychiatry.

[46] Jang Y, Chiriboga DA, Molinari V, Roh S, Park Y, Kwon S (2014). Telecounseling for the linguistically isolated: a pilot study with older Korean immigrants. Gerontologist.

[47] Ye J, Shim R, Lukaszewski T, Yun K, Kim SH, Rust G (2012). Telepsychiatry services for Korean immigrants. Telemed E-Health.

[48] Yeung A, Martinson MA, Baer L, Chen J, Clain A, Williams A (2016). The effectiveness of telepsychiatry-based culturally sensitive collaborative treatment for depressed Chinese American immigrants: a randomized controlled trial. J Clin Psychiatry.

[49] Pandya SP (2020). Online spiritual counseling mitigates immigration stress and promotes better marital adjustment of South Asian young dual-earner couples who emigrate to Western countries. Contemp Fam Ther.

[50] Mucic D (2008). International telepsychiatry: a study of patient acceptability. J Telemed Telecare.

[51] Mucic D (2011). Transcultural telepsychiatry and its impact on patient satisfaction. Eur Psychiatry.

[52] Zhu SH, Cummins SE, Wong S, Gamst AC, Tedeschi GJ, Reyes-Nocon J (2012). The effects of a multilingual telephone quitline for Asian smokers: a randomized controlled trial. JNCI J Natl Cancer Inst.

[53] Wetter DW, Mazas C, Daza P, Nguyen L, Fouladi RT, Li Y (2007). Reaching and treating Spanish-speaking smokers through the national cancer institute’s cancer information service: a randomized controlled trial. Cancer.

[54] Serafini RA, Powell SK, Frere JJ, Saali A, Krystal HL, Kumar V (2021). Psychological distress in the face of a pandemic: an observational study characterizing the impact of COVID-19 on immigrant outpatient mental health. Psychiatry Res.

[55] Walker R, Koh L, Wollersheim D, Liamputtong P (2014). Social connectedness and mobile phone use among refugee women in Australia. Health Soc Care Commun.

[56] Wollersheim D, Koh L, Walker R, Liamputtong P (2013). Constant connections: piloting a mobile phone-based peer support program for Nuer (southern Sudanese) women. Aust J Prim Health.

[57] Zehetmair C, Nagy E, Leetz C, Cranz A, Kindermann D, Reddemann L (2020). Self-practice of stabilizing and guided imagery techniques for traumatized refugees via digital audio files: qualitative study. J Med Internet Res.

[58] Chiu T, Marziali E, Colantonio A, Carswell A, Gruneir M, Tang M (2009). Internet-Based caregiver support for Chinese Canadians taking care of a family member with Alzheimer disease and related dementia. Can J Aging.

[59] Sirin S, Plass JL, Homer BD, Vatanartiran S, Tsai T (2018). Digital game-based education for Syrian refugee children: project hope. Vulnerable Child Youth Stud.

[60] Holmes EA, Ghaderi A, Eriksson E, Lauri KO, Kukacka OM, Mamish M (2017). ‘I can’t concentrate’: a feasibility study with young refugees in Sweden on developing science-driven interventions for intrusive memories related to trauma. Behav Cogn Psychother.

[61] Quackenbush DM, Krasner A (2012). Avatar therapy: where technology, symbols, culture, and connection collide. J Psychiatr Pract.

[62] Zheng P, Gray MJ (2014). Telehealth-based therapy connecting rural Mandarin-speaking traumatized clients with a Mandarin-speaking therapist. Clin Case Stud.

[63] Kayrouz R, Dear BF, Karin E, Gandy M, Fogliati VJ, Terides MD (2016). A pilot study of self-guided internet-delivered cognitive behavioural therapy for anxiety and depression among Arabs. Internet Interv Appl Inf Technol Ment Behav Health.

[64] Richards D, Richardson T (2012). Computer-based psychological treatments for depression: a systematic review and meta-analysis. Clin Psychol Rev.

[65] Di Carlo F, Sociali A, Picutti E, Pettorruso M, Vellante F, Verrastro V (2021). Telepsychiatry and other cutting-edge technologies in COVID-19 pandemic: bridging the distance in mental health assistance. Int J Clin Pract Esher.

[66] Andrade LH, Alonso J, Mneimneh Z, Wells JE, Al-Hamzawi A, Borges G (2014). Barriers to mental health treatment: results from the WHO world mental health surveys. Psychol Med.

[67] Guadagno L. Migrants and the COVID-19 pandemic: an initial analysis [Internet]. IOM; 2020. Available from: publications.iom.int/system/files/pdf/mrs-60.pdf

[68] Tirado-Morueta R, Aguaded-Gómez JI, Hernando-Gómez Á (2018). The socio-demographic divide in Internet usage moderated by digital literacy support. Technol Soc.

[69] Johnson S, Bacsu J, McIntosh T, Jeffery B, Novik N (2021). Competing challenges for immigrant seniors: social isolation and the pandemic. Healthc Manag Forum.

[70] Berthold SM, Libal K (2019). Refugees and asylum seekers: interdisciplinary and comparative perspectives.

[71] Ingleby D (2005). Forced migration and mental health [Internet].

[72] Taftaf R, Williams C (2020). Supporting refugee distance education: a review of the literature. Am J Distance Educ.

[73] Hoffmeyer-Zlotnik P. Country report: types of accommodation, Germany. Asylum information database, [Internet]. European Council of Refugees and Exiles.; 2022. Available from: https://asylumineurope.org/reports/country/germany/reception-conditions/housing/types-accommodation/

[74] Hassan H, Blackwood L (2021). (Mis)recognition in the therapeutic alliance: the experience of mental health interpreters working with refugees in U.K. clinical settings. Qual Health Res.

[75] O’Mara B, Monani D, Carey G (2021). Telehealth, COVID-19 and refugees and migrants in Australia: policy and related barriers and opportunities for more inclusive health and technology systems. Int J Health Policy Manag.

[76] Gajaria A, Conn DK, Madan R (2015). Telepsychiatry: effectiveness and feasibility. Smart Homecare Technol TeleHealth.

[77] Markowitz JC, Milrod B, Heckman TG, Bergman M, Amsalem D, Zalman H (2021). Psychotherapy at a distance. Am J Psychiatry.

[78] Connolly SL, Hogan TP, Shimada SL, Miller CJ (2021). Leveraging implementation science to understand factors influencing sustained use of mental health apps: a narrative review. J Technol Behav Sci.

[79] Harst L, Lantzsch H, Scheibe M (2019). Theories predicting end-user acceptance of telemedicine use: systematic review. J Med Internet Res.

[80] Mohr DC, Weingardt KR, Reddy M, Schueller SM (2017). Three problems with current digital mental health research … and three things we can do about them. Psychiatr Serv.

[81] Spanhel K, Balci S, Feldhahn F, Bengel J, Baumeister H, Sander LB (2021). Cultural adaptation of internet- and mobile-based interventions for mental disorders: a systematic review. Npj Digit Med.

[82] Hassan A, Sharif K (2019). Efficacy of telepsychiatry in refugee populations: a systematic review of the evidence. Cureus.

[83] Cowan KE, McKean AJ, Gentry MT, Hilty DM (2019). Barriers to use of telepsychiatry: clinicians as gatekeepers. Mayo Clin Proc.

[84] Horvath AO, Luborsky L (1993). The role of the therapeutic alliance in psychotherapy. J Consult Clin Psychol.

[85] Johansson H, Eklund M (2003). Patients’ opinion on what constitutes good psychiatric care. Scand J Caring Sci.

[86] Comas-Díaz L, Goodheart CD, Kazdin AE, Sternberg RJ (2006). Cultural variation in the therapeutic relationship. Evidence-based psychotherapy: where practice and research meet [Internet].

[87] Kuo BCH, Arthur N (2018). Refugee-serving multicultural therapy practicum: an example of a culture-infused, service-based training program. Counselling in cultural contexts [Internet].

[88] Rashid M (2020). Virtual care: do virtual visits risk leaving some people behind?.

[89] Vasquez MJT (2007). Cultural difference and the therapeutic alliance: an evidence-based analysis. Am Psychol.

[90] Strand M, Gammon D, Eng LS, Ruland C (2017). Exploring working relationships in mental health care via an E-recovery portal: qualitative study on the experiences of service users and health providers. JMIR Ment Health.

[91] Feijt MA, de Kort YAW, Westerink JHDM (2021). Assessing professionals’ adoption readiness for e-mental health: development and validation of the e-mental health adoption readiness scale. J Med Internet Res.

