# Correction: Access to Virtual Mental Healthcare and Support for Refugee and Immigrant Groups: A Scoping Review

**DOI:** 10.1007/s10903-023-01525-x

**Published:** 2023-07-31

**Authors:** Michaela Hynie, Anna Oda, Michael Calaresu, Ben C. H. Kuo, Nicole Ives, Annie Jaimes, Nimo Bokore, Carolyn Beukeboom, Farah Ahmad, Neil Arya, Rachel Samuel, Safwath Farooqui, Jenna‑Louise Palmer‑Dyer, Kwame McKenzie

**Affiliations:** 1 Department of Psychology, York University, Toronto, Canada; 2 Centre for Refugee Studies, York University, 4700 Keele St, Toronto, ON M3J1P3 Canada; 3 Department of Psychology, University of Alberta, Edmonton, Canada; 4 Department of Psychology, University of Windsor, Windsor, Canada; 5 School of Social Work, McGill University, Montreal, Canada; 6 Department of Psychology, University of Quebec in Montreal, Montreal, Canada; 7 School of Social Work, Carleton University, Ottawa, Canada; 8 School of Nursing, Western University, London, Canada; 9 School of Health Policy and Management, York University, Toronto, Canada; 10 Department of Family Medicine, McMaster University, Hamilton, Canada; 11 Counseling Psychology, Yorkville University, Fredericton, Canada; 12 Wellesley Institute, Toronto, Canada; 13 Division of Health Equity, CAMH, Toronto, Canada; 14 Department of Psychiatry, University of Toronto, Toronto, Canada

**Correction to: Journal of Immigrant and Minority Health**

10.1007/s10903-023-01521-1

The original version of this article unfortunately contained a typo in co-author name.

The co-author’s name should be spelled as Carolyn Beukeboom instead it was published incorrectly as Caroline Beukeboom.

The original article has been corrected.

